# Prognostic Implications of LRP1B and Its Relationship with the Tumor-Infiltrating Immune Cells in Gastric Cancer

**DOI:** 10.3390/cancers15245759

**Published:** 2023-12-08

**Authors:** Rui Wang, Guangtao Zhang, Xiaohong Zhu, Yan Xu, Nida Cao, Zhaoyan Li, Chen Han, Mengmeng Qin, Yumiao Shen, Jiahuan Dong, Fangqi Ma, Aiguang Zhao

**Affiliations:** 1 Department of Oncology, Longhua Hospital, Shanghai University of Traditional Chinese Medicine, Shanghai 200032, China; 18501657507@163.com (R.W.); guang11258@163.com (G.Z.); x777zz@163.com (X.Z.); plple1111@126.com (Y.X.); amandatsao77@163.com (N.C.);; 2 Department of Gastroenterology, Shanghai Municipal Hospital of Traditional Chinese Medicine, Shanghai University of Traditional Chinese Medicine, Shanghai 200071, China; 3 Department of Traditional Chinese Medicine, School of Medicine Affiliated Ruijin Hospital, Shanghai Jiao Tong University, Shanghai 200025, China

**Keywords:** gastric cancer, LRP1B, prognosis, tumor-infiltrating immune cells, immunochemistry

## Abstract

**Simple Summary:**

Gastric cancer (GC) has a poor prognosis, as it has often grown into an advanced stage when diagnosed. Genetic testing is crucial for establishing a treatment plan. In this study, next-generation sequencing (NGS) was performed to discover that LRP1B mutation was associated with a poor prognosis in GC. Through layer-by-layer research, it was found that LRP1B expression in GC was accompanied by a higher-level infiltration of CD4^+^ T, CD8^+^ T cells, and CD86/CD163. It is concluded that LRP1B expression can stimulate tumor immune cell infiltration, thus bringing clinical benefits to GC patients.

**Abstract:**

Background: Recent studies have shown that low-density lipoprotein receptor-related protein 1b (LRP1B), as a potential tumor suppressor, is implicated in the response to immunotherapy. The frequency of LRP1B mutation gene is high in many cancers, but its role in gastric cancer (GC) has not been determined. Methods: The prognostic value of LRP1B mutation in a cohort containing 100 patients having received radical gastrectomy for stage II–III GC was explored. By analyzing the data of LRP1B mRNA, the risk score of differentially expressed genes (DEGs) between LRP1B mutation-type and wild-type was constructed based on the TCGA-STAD cohort. The infiltration of tumor immune cells was evaluated by the CYBERSORT algorithm and verified by immunohistochemistry. Results: LRP1B gene mutation was an independent risk factor for disease-free survival (DFS) in GC patients (HR = 2.57, 95% CI: 1.28–5.14, *p* = 0.008). The Kaplan–Meier curve demonstrated a shorter survival time in high-risk patients stratified according to risk score (*p* < 0.0001). CYBERSORT analysis showed that the DEGs were mainly concentrated in CD4^+^ T cells and macrophages. TIMER analysis suggested that LRP1B expression was associated with the infiltration of CD4^+^ T cells and macrophages. Immunohistochemistry demonstrated that LRP1B was expressed in the tumor cells (TCs) and immune cells in 16/89 and 26/89 of the cohort, respectively. LRP1B-positive TCs were associated with higher levels of CD4^+^ T cells, CD8^+^ T cells, and CD86/CD163 (*p* < 0.05). Multivariate analysis showed that LRP1B-positive TCs represented an independent protective factor of DFS in GC patients (HR = 0.43, 95% CI: 0.10–0.93, *p* = 0.042). Conclusions: LRP1B has a high prognostic value in GC. LRP1B may stimulate tumor immune cell infiltration to provide GC patients with survival benefits.

## 1. Introduction

Gastric cancer (GC) ranks the fifth most common malignancy and the third leading cause of cancer death worldwide [1]. GC patients in the same TNM stage may demonstrate varying clinical outcomes, indicating the heterogeneity of cancer cell activity and tumor microenvironment (TME) of GC [2,3]. Therefore, individualized treatment is expected to improve the prognosis [4]. Next-generation sequencing (NGS) helps to identify the diagnostic and prognostic markers for tumors [5]. TME is closely correlated with tumorigenesis and progression [6,7]. In the TME, tumor-infiltrating immune cells (TIIs) act to inhibit or promote tumor progression. An antitumor immune response can only be propped up under a good cooperation of TIIs in the immune system, such as CD4^+^ T cells, CD8^+^ T cells, and macrophages [8]. A multi-center study has shown that immunotherapy is more effective in GC patients harboring LRP1B-P/LP (low-density lipoprotein receptor-related protein 1B—pathogenic/likely pathogenic) mutation [9]. However, the relationship between LRP1B and TIIs in GC remains unclear.

LRP1B is located on chromosome 2q, containing >91 exons and spanning 5 million bases. As a member of the low-density lipoprotein family, LRP1B has a molecular weight greater than 520 KDa [10]. A study has shown that LRP1B is regulated by DNA methylation to suppress GC development [11]. Some studies have confirmed that LRP1B is mutated or inactivated in many solid tumors. [12,13,14,15]. However, the role of LRP1B in GC needs further investigations.

In this study, LRP1B, as a prognostic gene for GC, was identified from the high-frequency mutation genes detected by previous NGS [16]. Furthermore, CYBERSORT and TIMER databases were used to analyze the TIIs in abnormal infiltration. Finally, the relationship between LRP1B expression and TII infiltration was evaluated through immunohistochemistry. The prognostic value of LRP1B in GC was investigated.

## 2. Materials and Methods

### 2.1. Patients and Samples

Recruited into this prospective study were a total of 100 patients admitted to Longhua Hospital, Shanghai University of Traditional Chinese Medicine and Renji Hospital, Shanghai Jiaotong University School of Medicine between 21 March 2018 and 3 September 2021. The study was approved by the ethics committee of Longhua Hospital (2018LCSY014) and Renji Hospital (2018-120). Written informed consent was provided from each participant.

Included were patients who (1) had pathologically confirmed GC and had undergone R0 section by radical gastrectomy; (2) were in TNM stages II, III according to the 8th American Joint Committee on Cancer; (3) were aged ≥18 years old; (4) had Karnofsky’s performance score ≥70; (4) had given informed consent. The exclusion criteria included stage I, IV; pre-gastrectomy treatment, and double or multiple cancers; pregnant women, lactating women, or mental illness. The included patients were treated with adjuvant chemotherapy according to the National Comprehensive Cancer Network guidelines. Their clinicopathological data were collected, and the NGS including 450-gene panel was performed.

The patients were routinely followed up (every 3 months during the first 2 years after surgery, every 6 months during the additional 3 years). Using laboratory tests, chest radiography and enhanced CT of the upper and lower abdomen were conducted. Disease-free survival (DFS) was defined as the time span from the date of enrollment to the first occurrence of recurrence, metastasis, or death. Overall survival (OS) was defined as the period from the date of enrollment to the date of death or final follow-up. The follow-up was censored on 30 December 2021.

### 2.2. Construction of a Prognostic Model

The clinicopathological data of 100 patients were collected, including gender, age, family history of cancer, tumor location, depth of invasion, lymph node metastasis, pathological stage, histological type, Lauren type, adjuvant chemotherapy cycle, and immunohistochemical indicators. Based on NGS detection of 450 genes, the high-frequency mutation genes were selected as *TP53*, *LRP1B*, *ARID1A*, *FAT4*, *RNF43*, *KMT2D*, *FAT3*, *ERBB3*, *CLI3*, *PIK3CA*, *PTEN*, *TFE3*, *ACVR2A*, *TGFBR2*, *KRAS*, *ERBB2*, *TGFBR1*, *HNF1A*, *RAD50*, *FGFR*, *RNF43*, *PIK3CA*. Tumor mutation burden (TMB) and microsatellite status estimation methods were estimated, as stated previously [16]. The above indicators were dichotomous or multiclass variables.

Least absolute shrinkage and selection operator (LASSO) regression analysis was performed with the “glmnet” R package to select the most significant variables related with prognosis, which were further subjected to multivariate Cox regression to construct receiver operating characteristic (ROC) curves using the “survivalROC” R package. Next, a nomogram model was developed by the “rms” R packages. The predictive accuracy of the nomogram was assessed through calibration curves and C-index.

### 2.3. Analysis Based on Public Datasets

LRP1B was identified as a prognostic gene of GC. The genomic information of LRP1B was searched in the ClinVar (https://www.ncbi.nlm.nih.gov/clinvar/ (accessed on 16 October 2021)) database and visualized in UCSC (https://geneme.ucsc.edu/ (accessed on 16 October 2021)). Next, the transcriptome RNA-Seq raw counts of LRP1B in GC patients were downloaded from TCGA-STAD (https://portal.gdc.cancer.gov (accessed on 20 October 2021)), a public database. Corresponding clinical information was extracted. The downloaded samples were divided into LRP1B mutation-type and wild-type. The “DESeq2” R package was used to screen differentially expressed genes (DEGs) in LRP1B mutation-type and wild-type, with |Fold Change| ≥ 2 and *p*-value ≤ 0.05 as the cutoff value. The significant DEGs were screened out using univariant Cox regression and LASSO analyses. The DEGs filtered by LASSO analysis were subjected to the multivariate Cox regression analysis, and Cox regression coefficients were extracted. The risk score of each patient was calculated by combining gene expression level and Cox regression coefficients, as shown in the following formula: risk score = βmRNA1 × exprmRNA1 + βmRNA2 × exprmRNA2 + … + βmRNAn × exprmRNAn, where expermRNA indicates the expression level of DEGs, and βmRNA indicates the regression coefficient of DEGs in the multivariate regression analysis [17,18]. Patients were divided into high-risk and low-risk groups according to the median score. The Kaplan–Meier (K-M) survival curve between the two groups was constructed. Then the 1-, 3-, and 5-year ROC curves were drawn based on the risk score. The abundances and proportions of 22 immune infiltrating cells in LRP1B mutation-type and wild-type were calculated by the “CYBERSORT” algorithm in R, according to downloaded RNA-Seq sequencing data. The TIMER public database (https://cistrome.shinyapps.io/timer/ (accessed on 23 October 2021)) was also used to explore LRP1B mutation and expression in the infiltrating immune cell.

### 2.4. Immunohistochemistry

Tissue sections were obtained from 89 of 100 GC patients. Formalin-fixed paraffin-embedded (FFPE) tissue sections were deparaffinized in xylene and graded ethanol solutions. Next, antigen recovery was carried out in the immunohistochemistry pretreatment system (PT Link, Dako, Copenhagen, Denmark). The sections were then cooled at room temperature, washed in PBS, and incubated with primary antibodies, anti-LRP1B (HPA069094, dilution: 1:800, Atlas Antibodies, Voltavägen, Bromma, Sweden), anti-CD4 (ZA0519, ZSGB-BIO, Beijing, China), anti-CD8 (Mab0021, MXB, Fuzhou, China), anti-CD86 (91882S, dilution: 1:300, CST, Danvers, MA, USA), anti-CD163 (Mab0206, MXB, China), anti-CD25 (ab231441, dilution: 1:200, Abcam, Cambridge, UK) at room temperature for 55 min. After the primary antibodies were removed, PBS buffer solution was used to wash the sections three times, followed by incubation with the secondary antibodies (Goat Anti-Rabbit, 41293161, Dako, Copenhagen, Denmark) at room temperature for 20 min. After dehydration and sealing, the sections were stained with DAB and hematoxylin.

### 2.5. Assessment of Immunostaining

Immunostaining images were reviewed and signed off on by two pathologists at 20× magnification who were blinded to clinical outcome. The expression of LRP1B was observed in tumor cells (TCs) and immune cells (ICs). LRP1B-positive TCs were those with membranes stained brown. LRP1B-positive ICs were those with membranes stained at any intensity. The fields were defined by TIIs (CD4, CD8, CD86, CD163, CD25) stained in the tumor area of GC tissue samples. TII-positive samples were those with membrane stained at any intensity. First, the fields of dense TIIs in the tumor tissue samples were defined at a low magnification (10× magnification), and 5 fields with the highest density (positive cells/mm^2^) were observed at a high magnification (20× magnification). The mean count in the five fields was determined as the final density of the TIIs. The median density was taken as the cut-off value to distinguish high from low immune cell infiltration [19,20].

### 2.6. Statistical Analysis

The associations between LRP1B, CD4, CD8, CD86, CD163, CD25 levels and clinicopathological parameters were illustrated using the Chi square test or Fisher’s exact test. The degree of correlation was further evaluated through the binary logistic regression analysis. The above analyses were performed on SPSS (version 26.0) and plotted by GraphPad Prism (version 8.0.1). The K-M curves were plotted by log-rank tests. *p*-values less than 0.05 were considered statistically significant.

## 3. Results

### 3.1. Clinicopathological Characteristics of Patients

A total of 100 patients with GC were enrolled, including 15 (15%) in stage II and 85 (85%) in stage III. Of them, 72 (72%) patients were aged 60 years or older, 75 (75%) patients were male, 28 (28%) patients had a family history of cancer, 71 (71%) patients had completed adjuvant chemotherapy. The median follow-up time was 27.7 months (range 2.2–43.7 months). At the end of follow-up, 44% (44/100) of patients showed recurrence and metastasis. The clinicopathological characteristics of the included patients are summarized in Table 1. In addition, the indicators incorporated into LASSO regression analysis were as follows: HER-2, Ki67, p53, stat3, PTBP3, RUFY3, bcl2, PD-L1, TMB, and microsatellite status. The top 20 high-frequency mutation genes were classified into mutation-type and wild-type. The above information is detailed in Supplementary Table S1.

### 3.2. Correlation between LRP1B and Prognosis of GC

**Figure 1:**
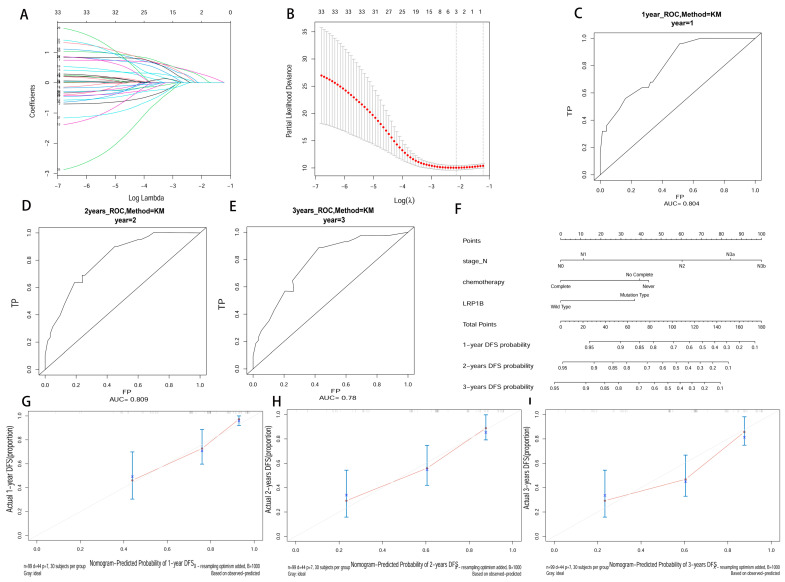
Constructing a nomogram integrating LRP1B and clinical factors. (**A**) The Lasso coefficient profiles of survival-related variables. (**B**) Tenfold cross-validation for turning parameter (lambda) selection in the LASSO model based on the minimum criteria for DFS. (**C**–**E**) ROC curve analysis at 1, 2, and 3 years of patients (AUC = 0.804, 0.809, 0.078). (**F**) Nomogram to predict disease-free survival probability at 1, 2, and 3 years. (**G**–**I**) Calibration curve for the nomogram predicting 1-, 2-, and 3-year disease-free survival.

In the LASSO regression analysis, three biomarkers related to DFS in GC were screened out of the 37 variables mentioned above, based on the lowest lambda values (Figure 1A,B). Then, the multivariate Cox analysis identified LRP1B, pathological-N stage, and adjuvant chemotherapy cycle as independent prognostic factors of DFS in GC (Table 2). Compared with the LRP1B wild-type, the mutation-type was associated with a 2.57 times higher risk of recurrence or metastasis (95% CI: 1.28–5.14, *p* = 0.008). The performance of multivariate Cox analysis was assessed by ROC curve. The areas under ROC (AUCs) of 1-, 2-, and 3-year DFSs were 0.804, 0.809, and 0.780, respectively (Figure 1C–E). Next, a nomogram including LRP1B, pathological-N stage, and adjuvant chemotherapy cycle was established to predict the probabilities of 1-, 2-, and 3-year DFSs in GC patients (Figure 1F). The calibration plots demonstrated a good agreement between the actual and predicted probabilities (Figure 1G–I). The C-index for the nomogram was 0.741, which indicated its strong discriminative ability.

### 3.3. LRP1B_mRNA Expression from TCGA

**Figure 2:**
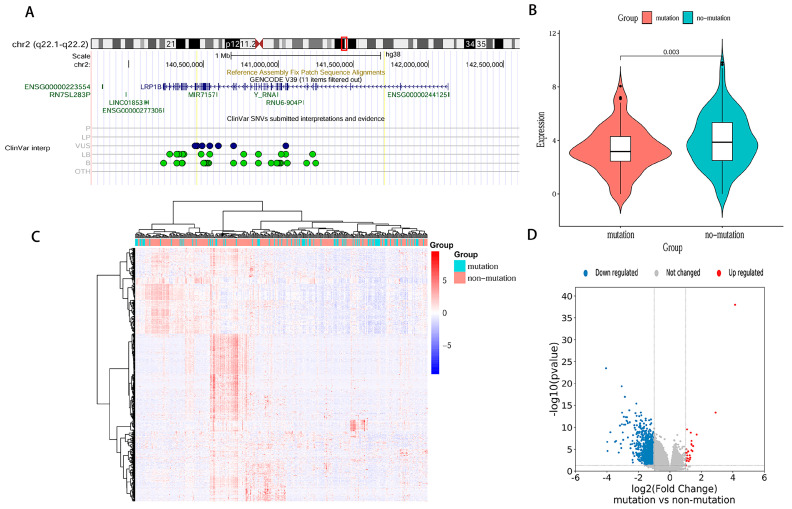
Schematic diagram of LRP1B and differential expression analysis of TCGA-STAD based on LRP1B status. (**A**) The LRP1B genome information map. (**B**) mRNA expression in TCGA-STAD patients with LRP1B^WT^ and LRP1B^MUT^. (**C**) Heatmap plot of mRNA expression in TCGA-STAD patients with LRP1B^WT^ and LRP1B^MUT^. (**D**) Volcano plot of differentially expressed genes identified in TCGA-STAD patients with LRP1B^WT^ and LRP1B^MUT^ (DEGs: differentially expressed genes; WT: wild-type; MUT: mutation-type; The blue and green dots in Figure 2A represented different sites).

**Figure 3:**
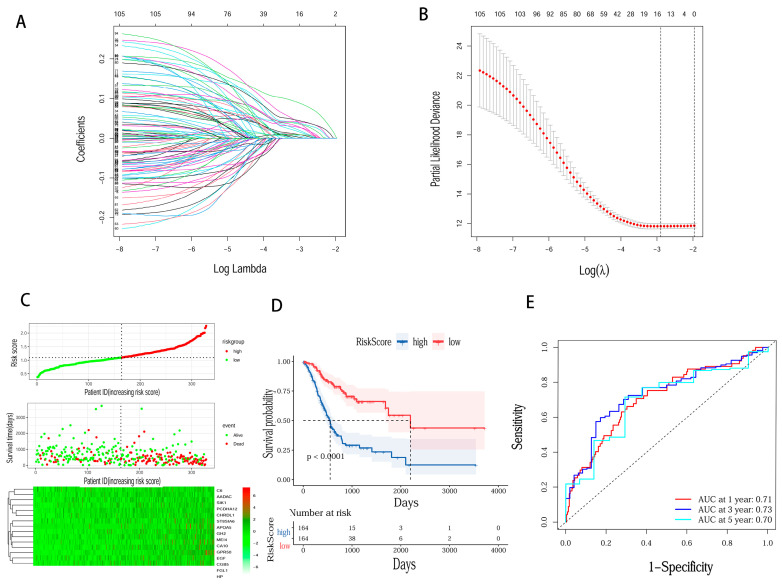
Development of the prognostic signature based on 15 DEGs identified in TCGA-STAD patients with LRP1B^WT^ and LRP1B^MUT^. (**A**) The Lasso coefficient profiles of OS-related DEGs; (**B**) selection of 15 DEGs in the LASSO model based on the minimum criteria for prognosis. (**C**) Risk score distribution, survival status, and 15 DEG expression profiles for patients in high-risk and low-risk groups. (**D**) Difference in OS between high-risk and low-risk groups of the TCGA-STAD patients. (**E**) Time-dependent ROC curve analysis at 1, 2, and 3 years (DEGs: differentially expressed genes; WT: wild-type; MUT: mutation-type; OS: overall survival).

**Figure 4:**
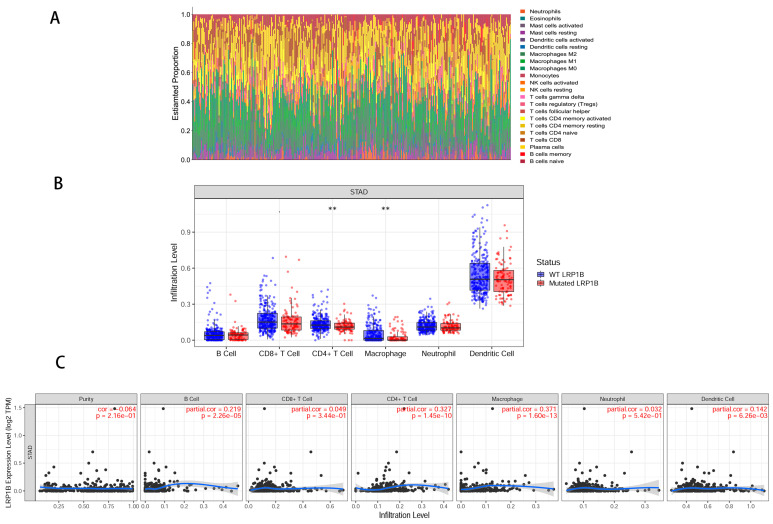
Immune infiltration landscape in LRP1B molecule. (**A**) Relative proportion of immune cell infiltrations in LRP1BWT and LRP1BMUT patients with TCGA-STAD. (**B**) Infiltration abundance of LRP1BWT and LRP1BMUT in 6 immune cells. (**C**) The relationship between the expression level of LRP1B and 6 kinds of immune infiltrating cells (WT: wild-type; MUT: mutation-type; ** *p <* 0.01).

The genomic map of LRP1B is shown in Figure 2A. The gene is located on chromosome 2q22.1–q22.2 and has two known mutation sites: rs6747180 and rs12614785. A total of 328 samples with mRNA expression profiles and clinical data were obtained from the TCGA-STAD database. The STAD patients were separated into LRP1B mutation-type (86 samples) and wild-type (242 samples) groups and DEGs were explored (Figure 2B,C). In total, 1022 downregulated genes and 26 upregulated genes were identified (Figure 2D). The clinical data of patients in TCGA-STAD are shown in Table 3 (only the OS of TCGA-STAD can be obtained). The univariate Cox analysis showed that the 108 DEGs were related to OS in TCGA-STAD patients (*p* < 0.05) (Supplementary Figure S1). Then, 15 DEGs were identified as prognostic signatures by the LASSO regression analysis (Figure 3A,B). Finally, the expression and coefficient values of 15 DEGs were extracted to compute the risk score for each patient. Based on the median value, the patients in TCGA-STAD were separated into low-risk and high-risk groups (Figure 3C). The K-M curve demonstrated that high-risk patients had a shorter survival time than low-risk patients (*p* < 0.0001) (Figure 3D). The ROCs of risk score for 1-, 3-, and 5-year OS were 0.71, 0.73, and 0.70, respectively (Figure 3E). The DEGs between LRP1B mutation-type and wild-type were mainly enriched in CD4^+^ T cells and macrophages (Figure 4A). The results of TIMER analysis demonstrated a statistical difference between LRP1B mutation-type and wild-type in CD4^+^ T cells and macrophages. LRP1B mutation-type was accompanied by a lower-degree infiltration of CD4^+^ T cells and macrophages (*p* < 0.01) (Figure 4B). TIMER analysis indicated that LRP1B expression was significantly correlated with the infiltration of CD4^+^T cells (Cor = 0.327) and macrophages (Cor = 0.371) (Figure 4C).

### 3.4. LRP1B Expression and Clinicopathological Characteristics

**Figure 5:**
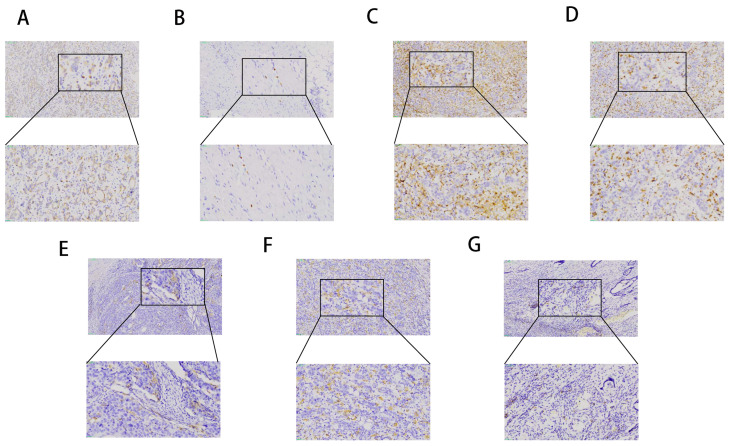
Representative immunohistochemical staining of LRP1B and tumor-infiltrating immune cells in human gastric cancer tissue. (**A**) LRP1B expression on TCs (magnifications ×100; magnifications ×200). (**B**) LRP1B expression on ICs (magnifications ×100; magnifications ×200). (**C**) CD4^+^ TII expression on TCs (magnifications ×100; magnifications ×200). (**D**) CD8+ TII expression on TCs (magnifications ×100; magnifications ×200). (**E**) CD86^+^ TII expression on TCs (magnifications ×100; magnifications ×200). (**F**) CD163^+^ TII expression on TCs (magnifications ×100; magnifications ×200). (**G**) CD25^+^ TII expression on TCs (magnifications ×100; magnifications ×200) (TCs: tumor cells; ICs: immune cells; TIIs: tumor-infiltrating immune cells).

Immunohistochemistry was performed on GC tissues of 89 patients to determine the relationship between LRP1B protein expression and the level of TIIs: CD4^+^ T cells, CD8+ T cells, M1 macrophages, M2 macrophages, and Treg cells. The results showed that LRP1B protein was expressed in TC membranes and IC membranes (Figure 5A,B). LRP1B was positively expressed in the ICs of 28 cases (31.46%), and the TCs of only 16 cases (17.98%). The relationship of LRP1B expression in TCs and ICs with clinicopathological characteristics is shown in Table 4. Positive LRP1B expression in TCs was correlated with PD-L1, but not with age, tumor location, histological type, Lauren type, TNM stage, HER-2, TMB, or microsatellite status. No correlation showed up between positive LRP1B expression in ICs and any of the clinicopathological characteristics.

### 3.5. TII Expression in GC Tissues

Lymphoid markers CD4^+^ and CD8^+^, macrophage markers CD86^+^ and CD163^+^, and regulatory T cell markers CD25^+^ were analyzed by immunohistochemistry. Each cell subset was manually counted. The median density of CD4^+^ TIIs in the tumor area of GC tissue was 30.8 (range, 1–326) cells/HPF (high-power field); that of CD8^+^ TIIs was 49.6 (2–523) cells/HPF; that of CD86^+^ TIIs was 0.4 (1–43) cells/HPF; that of CD163^+^ TIIs was 19.4 (1–184) cells/HPF; and that of CD25^+^ TIIs was 0 (0–11) cells/HPF (Supplementary Table S2). High expression was defined as the average number of TIIs higher than the median, and vice versa. Representative stains of these immune markers are presented in Figure 5C–G.

### 3.6. Association of LRP1B^+^TCs with High Levels of CD4^+^ TIIs, CD8^+^ TIIs, and CD86/CD163

**Figure 6:**
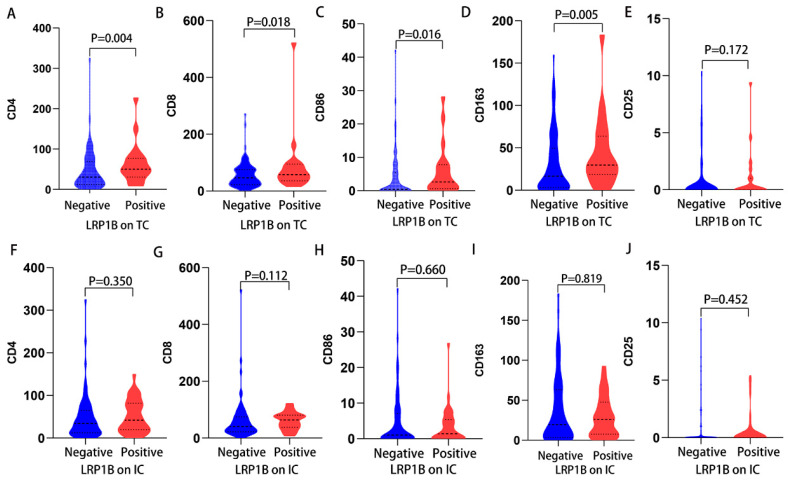
The relationship between LRP1B expression on TCs/ICs and TIIs in human gastric cancer tissue. (**A**) LRP1B expression on TCs with CD4^+^ TIIs. (**B**) LRP1B expression on TCs with CD8^+^ TIIs. (**C**) LRP1B expression on TCs with CD86^+^TIIs. (**D**) LRP1B expression on TCs with CD163^+^TIIs. (**E**) LRP1B expression on TCs with CD25^+^TIIs. (**F**) LRP1B expression on ICs with CD4^+^ TIIs. (**G**) LRP1B expression on ICs with CD8^+^ TIIs. (**H**) LRP1B expression on ICs with CD86^+^ TIIs. (**I**) LRP1B expression on ICs with CD163^+^ TIIs. (**J**) LRP1B expression on ICs with CD25^+^ TIIs (TCs: tumor cells; ICs: immune cells; TIIs: tumor-infiltrating immune cells).

As shown in Figure 6A–E, positive LRP1B expression in TCs showed a correlation with high levels of CD4^+^ TIIs (*p* = 0.004), CD8^+^ TIIs (*p* = 0.018), CD86^+^ TIIs (*p* = 0.016), and CD163^+^ TIIs (*p* = 0.005). The connection between LRP1B expression in ICs and the five types of TIIs was not established (Figure 6F–J). Positive LRP1B expression in TCs, compared to its negative expression, was associated with a higher CD86/CD163 ratio (*p* = 0.014), but this association was not observed in ICs (Supplementary Figure S2A–D). No connection appeared between LRP1B expression and CD4/CD8.

### 3.7. Prognostic Significance of LRP1B, TIIs Status for DFS and OS

**Figure 7:**
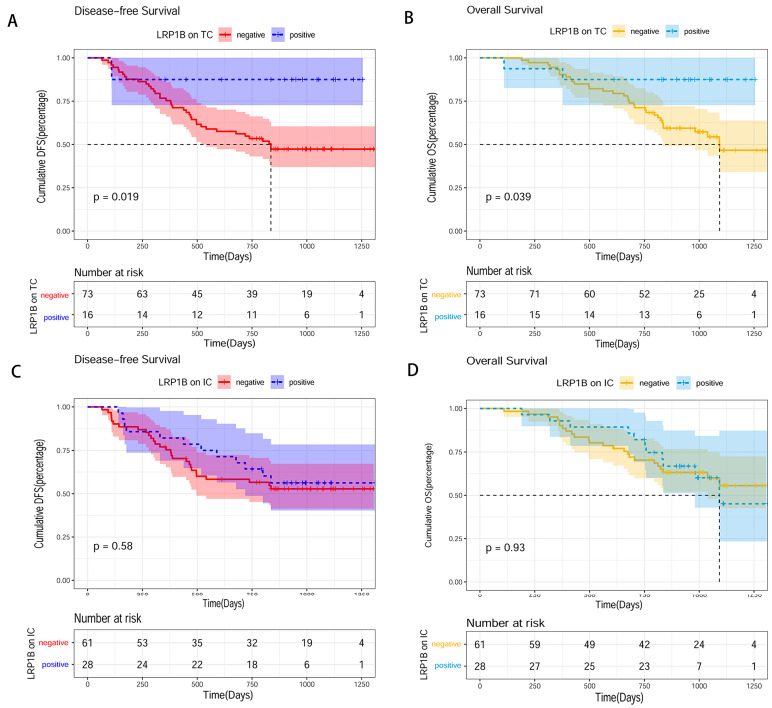
Kaplan–Meier survival analysis of LRP1B expression on TCs/ICs in human gastric cancer tissue. (**A**) DFS of LRP1B expression on TCs. (**B**) OS of LRP1B expression on TCs. (**C**) DFS of LRP1B expression on ICs. (**D**) OS of LRP1B expression on ICs (TCs: tumor cells; ICs: immune cells; DFS: disease-free survival; OS: overall survival).

By 30 December 2021, the median follow-up time was 26.6 (range, 2.2–43.7) months. Among the 89 patients, 35 (39.33%) patients died and 40 (44.94%) experienced recurrence or metastasis. The K-M plots demonstrated that the LRP1B-positive TC group had longer DFS (*p* = 0.019) and OS (*p* = 0.016) than the negative group, and LRP1B expression in ICs was not related to prognosis (Figure 7A–D). The univariate Cox analysis determined that LRP1B-positive TCs, a higher ratio of CD4/CD8, PD-L1 positivity, and histological type were associated with longer DFS and OS (Supplementary Table S3). In the multivariate Cox analysis, a high ratio of CD4/CD8 was an independent factor associated with a favorable DFS (HR = 0.35, 95% CI: 0.16–0.74, *p* = 0.006) and OS (HR = 0.27, 95% CI: 0.12–0.65, *p* = 0.004). Positive LRP1B expression in TCs (HR = 0.43, 95% CI: 0.10–0.93, *p* = 0.042) was an independent protective indicator for a longer DFS (Table 5).

## 4. Discussion

In this study, NGS first discovered that patients with LRP1B mutation were more likely to have GC recurrence and metastasis. Further, public databases were used to filter out the DEGs between LRP1B mutation-type and wild-type, and their biological significance was analyzed. According to the DEGs, the risk score was calculated to predict the OS in each patient with GC (only the OS of GC can be found in the TCGA). The results further consolidated the prognostic value of the LRP1B gene in GC. Immune infiltration analysis showed that the DEGs were mainly concentrated in CD4^+^ T cells and macrophages. And the distribution of LRP1B mutation-type and wild-type showed differences in either CD4^+^ T cells or macrophages, implying a relationship between LRP1B expression and TIIs. TIMER analysis indicated that LRP1B expression was significantly correlated with the infiltration of CD4^+^ T cells and macrophages. In addition, immunohistochemistry on GC verified that evident relationships existed among LRP1B expression, TIIs, and prognosis. These relationships endow LRP1B with a high prognostic value for GC.

LRP1B is among the most aberrantly expressed genes in human tumors [21]. Our sequencing results demonstrated that LRP1B had a mutation rate of 23% (23/100) in GC, and 26% in the TCGA-STAD database, indicating that LRP1B takes on a similar profile in GC in eastern and western countries. LRP1B is frequently inactivated by genetic and epigenetic mechanisms, making it a putative tumor suppressor [22]. LRP1B deletion is associated with poor outcome in glioblastoma patients [23]. Our sequencing results indicated that LRP1B might undergo point mutations and deletion mutations. It is known that a deletion mutation can cause the loss of certain functions and reduce the expression of genes. Point mutation may ultimately dysregulate protein expression. In the present study, immunohistochemistry revealed that seven out of twenty-one LRP1B mutants (21/89) expressed LRP1B in the ICs, while only three out of twenty-one showed positive expression in the TCs. Statistical analysis showed no significant correlation between LRP1B mutation and expression. Previous studies have identified LRP1B as a tumor suppressor gene [10,11,13,22]. Deletion mutation can deprive LRP1B of its suppression effect, thus predicting a poor prognosis of GC. The molecular mechanism of LRP1B deletion in GC should be further elucidated by advanced basic experiments in the future.

This study indicated that LRP1B gene mutation was more prone to relapse or metastasis than wild-type GC patients. The nomogram, including the LRP1B gene, pathological-N stage, and adjuvant chemotherapy cycle, showed a good performance in predicting the DFS of GC, as shown by its C-index of 0.741 and calibration curves near the diagonal. Nomograms have become a popular tool to predict tumor prognosis [24,25,26]. A model absorbing genomics characteristics should be more meaningful for precision medicine. We explored the DEGs between LRP1B mutation-type and wild-type in the TCGA-STAD public database. The DEGs were mainly downregulated. Then, OS was taken as the endpoint for prognosis analysis, due to the lack of DFS data in the TCGA-STAD. The risk score was calculated using 15 DEGs screened by the LASSO analysis and based on dividing patients into high-risk and low-risk groups. The K-M curves indicated that low-risk patients had a longer OS (*p* < 0.001). Nevertheless, the prognostic significance and biological value of LRP1B should be further explored.

Johnson et al. have proposed that LRP1B alterations may increase mutational load and reshape the immune microenvironment [27]. A study suggests that LRP1B can be exploited to design immunotherapies [9]. Another study points out that LRP1B may serve as an endocytic receptor to facilitate the clearance of extracellular debris, thereby regulating the TME [28]. In the present study, through immune infiltration analysis, we found that the DEGs of LRP1B mutation-type and wild-type were mainly distributed in CD4^+^ T cells and macrophages. TIMER analysis suggested that LRP1B mutation-type differed from wild-type in the infiltration of CD4^+^ T cells and macrophages, and LRP1B expression was significantly correlated with the levels of CD4^+^ T cells and macrophages. The composition and proportion of TIIs vary in different cancers, playing critical roles in the occurrence and development of tumors [29]. Deficient CD4^+^ T cells repress the response of cytotoxic T lymphocytes (CTLs), while abundant CD4^+^ T cells can improve outcomes of cancer immunotherapy strategies [30]. CD8^+^ T lymphocytes are constantly activated to form CTLs, thus producing a persistent and effective anti-tumor immune response [31]. Tumor-associated macrophages differentiate into M1 and M2 types. M2 macrophages are tumor-promotive, while M1 macrophages are tumor-suppressive. CD86 and CD163 are used to identify M1 and M2 macrophages, respectively [32,33]. Tregs, marked by CD25, can increase the host’s immune tolerance through suppressing T cells or secreting immunosuppressive cytokines [34]. 

Our immunohistochemistry suggested that LRP1B was expressed in the membrane of TCs and ICs. In four patients, LRP1B was expressed in both TCs and ICs. One study has shown that LRP1B immunoreactivity is detected in the membrane and cytoplasm of breast cancer cells from 60 of 92 patients [35]. Another study has pointed that of 45/100 GC tissue specimens exhibited cytoplasmic localization of LRP1B [36]. LRP1B is a giant member of the LDLR protein family, which includes several structurally homologous cell surface receptors with a wide range of biological functions, from cargo transport to cell signaling [21]. As the LRP1B protein is hydrolyzed, parts of its fragments split in the membrane, and the remaining is transferred to the cytoplasm or nucleus [14]. Two studies have shown that CMTM6 and VISTA were expressed in both TCs and ICs in colorectal cancer [37] and breast cancer [38], respectively. Our study also found that LRP1B was expressed in the TC and IC membranes of GC tissues. These results indicate that LRP1B expression in TCs is positively correlated with a higher-degree infiltration of CD4^+^, CD8^+^, CD86^+^, and CD163^+^ immune cells, as well as a higher ratio of CD86/CD163 (*p* = 0.014). The survival analysis showed that the positive expression of LRP1B protein in TCs was an independent protective factor of DFS in GC (HR = 0.43, 95%: 0.10–0.93, *p* = 0.042), indicating that LRP1B expressed in TCs affected the proportion of TIIs in the TME. LRP1B expression in TCs stimulates the infiltration of CD4^+^ and CD8+ T cells and raises the ratio of CD86/CD163, which may be due to an increase in tumor immunogenicity to achieve immune activation and prevent disease progression. LRP1B is specifically expressed in TCs, making it a novel target to enhance anti-tumor responses and prolong the survival of GC patients.

One study has suggested that LRP1B mutation is associated with a higher TMB in hepatocellular carcinoma [39]. Another study remarks that LRP1B is a promising biomarker for predicting the efficacy of immunotherapy [28]. Two studies have confirmed the prognostic value of CD4/CD8 in solid tumors [40,41]. Another research work has demonstrated that the CD86/CD163 ratio may be used for individualized assessment of recurrence and mortality risk in colorectal cancer patients [42]. Therefore, we also conducted a prognostic analysis of CD4/CD8 and CD86/CD163 ratios. Our results also showed that a higher ratio of CD4/CD8 was an independent beneficial indicator for DFS and OS in GC patients. The CD4/CD8 ratio reflects not only adaptive immunity, but also the activation and inflammation in innate cells [43]. A positive change in lymphocyte ratio may enhance anti-tumor immune responses, thereby benefiting GC patients. Unprecedently, the present study delved into the relationship between LRP1B and TIIs. In the future, LRP1B immunohistochemistry should be performed on pathological tissues. LRP1B mutation status should be detected in the blood of GC patients to determine whether immunotherapy is needed. Alternatively, LRP1B-targeting drugs are expected to be developed. However, whether LRP1B can be applied in clinical practice like other immunotherapy biomarkers, such as TMB and MSI, still requires extensive research.

This study still has some limitations. First of all, the sample size was small. The nomogram was not verified internally and externally, and only 1000 instances of internal verification were resampled by the bootstrap method. Second, the multivariate Cox analysis confirmed that LRP1B mutation was an independent risk factor for DFS in GC. All patients received radical surgery for stage II–III gastric cancer, and few of them experienced OS. So no statistically significant difference was found between LRP1B and OS of GC in the survival analysis. As the follow-up is still under way, we will conduct a secondary analysis on the relationship between LRP1B and OS. The results of our study should also be validated in a well-designed prospective randomized controlled trial with a large sample size. Third, studies of LRP1B still have some limitations, in particular associated with its huge size. Our mechanism experiments on LRP1B ended in failure. We expected to manipulate LRP1B expression or mutation in vivo and in vitro by technical and methodological advances in the future. In addition, to explore the relationship between LRP1B protein expression and TIIs, immunofluorescence staining is the recommended method. However, co-staining of six proteins is difficult to carry out, so we only conducted immunohistochemistry experiments.

## 5. Conclusions

LRP1B gene mutation was an independent risk factor for DFS in GC patients. The DEGs between LRP1B mutant-type and wild-type could predict the OS of GC. Positive LRP1B expression in TCs was associated with higher levels of CD4^+^ T cells, CD8^+^ T cells, and CD86/CD163. LRP1B induced tumor immune cell infiltration, which may improve the outcomes of GC patients.

## Figures and Tables

**Table 1:** Clinicopathological characteristics in GC.

Parameters	N	%
Age		
<60	28	28.00
≥60	72	72.00
Gender		
Male	75	75.00
Female	25	25.00
Family history of tumor		
Yes	28	28.00
No	72	72.00
Tumor location		
EGJ/Cardia	17	17.00
Gastric body	26	26.00
Gastric antrum	57	57.00
p-TNM stage		
II	15	15.00
III	85	85.00
Tumor stage		
pT2	2	2.00
pT3	2	2.00
pT4a	67	67.00
pT4b	29	29.00
Lymph node		
pN0	16	16.00
pN1	12	12.00
pN2	16	16.00
pN3a	37	37.00
pN3b	19	19.00
Histological type		
Differentiated	76	76.00
Undifferentiated	4	4.00
Mixed	20	20.00
Lauren type		
Intestinal	54	54.00
Diffuse	31	31.00
Mixed	15	15.00
Chemotherapy cycle		
≥6	71	71.00
<6	24	24.00
No chemotherapy	5	5.00

GC, gastric cancer; N, number; EGJ, gastroesophageal junction; p, pathological.

**Table 2:** Multivariate analysis of DFS in patients with GC.

	HR (95% CI)		*p*-Value
Lymph node			0.001
pN0 vs. pN3b	0.077 (0.02–0.34)		0.001
pN1 vs. pN3b	0.103 (0.02–0.47)		0.003
pN2 vs. pN3b	0.363 (0.14–0.96)		0.041
pN3a vs. pN3b	0.673 (0.33–1.37)		0.277
Adjuvant chemotherapy cycle			0.013
<6 vs. ≥6	0.326 (0.10–1.16)		0.250
no vs. ≥6	0.891 (0.24–3.30)		0.699
LRP1B			
MUT vs. WT	2.57 (1.28–5.14)		0.008

DFS, disease-free survival; GC, gastric cancer; HR, hazard ratio; CI, confidence interval; p, pathological; no, no chemotherapy; MUT, mutation-type; WT, wild-type.

**Table 3:** Patient characteristics in TCGA database.

Parameters	Total, N (%)	LRP1B-MUT, N (%)	LRP1B-WT, N (%)	*p*-Value
	328 (100)	86 (26.22)	242 (73.78)	
Age	67 (58–72)	68 (59–73)	66 (57–72)	0.057
Gender				0.190
Female	119 (36.28)	26 (30.23)	93 (38.43)	
Male	209 (63.72)	60 (69.77)	149 (61.57)	
Race				0.700
Hispanic or Latino	5 (1.54)	1 (1.16)	4 (1.65)	
Not Hispanic or Latino	236 (71.95)	59 (68.60)	177 (73.14)	
NA	87 (26.51)	26 (30.24)	61 (25.21)	
Tumor location				0.071
Gastric body	81 (24.70)	20 (23.26)	61 (25.21)	
Cardia	79 (24.08)	14 (16.28)	65 (26.86)	
Gastric fundus	40 (12.19	9 (10.46)	31 (12.81)	
Gastric antrum	118 (35.98)	40 (46.51)	78 (32.23)	
Whole stomach	10 (3.05)	3 (3.49)	7 (2.89)	
p-TNM stage				0.440
I	43 (13.11)	8 (9.30)	35 (14.46)	
II	109 (33.23)	27 (31.40)	82 (33.88)	
III	144 (43.90)	40 (46.51)	104 (42.98)	
IV	32 (9.76)	11 (12.79)	21 (8.68)	

TCGA, The Cancer Genome Atlas; MUT, mutation-type; WT, wild-type; N, number; NA, not available; p, pathological.

**Table 4:** LRP1B on tumor cells, immune cells, and clinicopathological characteristics in GC.

Parameters	LRP1B on TCs			LRP1B on ICs		
	Negative N (%)	Positive N (%)	*p*-Value	Negative N (%)	Positive N (%)	*p*-Value
Total	73 (82.02)	16 (17.98)		61 (68.54)	28 (31.46)	
Age			0.217 *			0.777
<60 years	22 (30.14)	2 (11.50)		17 (27.87)	7 (25.00)	
≥60 years	51 (69.86)	14 (88.50)		44 (72.13)	21 (75.00)	
Tumor location			0.138 *			0.218 *
EGJ/cardia	6 (8.22)	5 (31.25)		5 (8.20)	6 (21.44)	
Gastric body	21 (28.77)	5 (31.25)		18 (29.51)	8 (28.57)	
Gastric antrum	46 (63.01)	6 (37.50)		38 (70.49)	14 (49.99)	
Histological type			0.669 *			0.160 *
Differentiated	55 (75.34)	13(81.25)		43 (70.49)	25 (89.29)	
Undifferentiated	3 (4.11)	1 (3.84)		4 (6.56)	0	
Mixed	15 (20.55)	2 (6.25)		14 (22.95)	3 (10.71)	
Lauren type			0.861 *			0.100 *
Intestinal	39 (53.42)	10 (62.50)		29 (47.54)	20 (71.43)	
Diffuse	24 (32.88)	4 (25.00)		23 (37.70)	5 (17.86)	
Mixed	10 (13.70)	2 (12.50)		9 (14.76)	3 (10.71)	
p-TNM stage			1.000 *			0.355 *
II	12 (16.44)	2 (12.50)		8 (13.11)	6 (21.43)	
III	61 (83.56)	14 (87.50)		53 (86.89)	22 (78.57)	
HER2			0.449 *			1.000 *
HER2-	61 (83.56)	15 (93.75)		52 (85.25)	24 (85.71)	
HER2+	12 (16.44)	1 (6.25)		9 (14.75)	4 (14.29)	
PD-L1			0.026			0.209
PD-L1-	36 (49.32)	3 (18.75)		24 (39.34)	15 (53.57)	
PD-L1+	37 (50.68)	13 (81.25)		37 (60.66)	13 (46.43)	
TMB			0.192			0.952
TMB-L	47 (64.38)	13 (81.25)		41 (67.21)	19 (67.86)	
TMB-H	26 (35.62)	3 (18.75)		20 (32.79)	9 (32.14)	
Microsatellite status			0.198 *		0.495 *	
MSS	63 (86.30)	16 (100)		53 (86.89)	26 (92.86)	
MSI-H	10 (13.70)	0		8 (13.11)	2 (7.14)	

TCs, tumor cells; ICs, immune cells; N, number; EGJ, gastroesophageal junction; p, pathological; HER2, human epidermal growth factor receptor 2; PD-L1, programmed cell death ligand 1; TMB, tumor mutation burden; L, low; H, high; MSS, microsatellite stability; MSI, microsatellite instability. *, Fisher’s precision probability test.

**Table 5:** Multivariate analysis for patients with GC.

Parameters	DFS		OS	
	HR (95% CI)	*p*-Value	HR (95% CI)	*p*-Value
Age				
<60	1		1	
≥60	0.96 (0.47–1.97)	0.911	1.29 (0.58–2.89)	0.534
Histological type		0.031		0.068
Differentiated	1		1	
Undifferentiated	1.08 (0.47–2.46)	0.856	0.82 (0.36–1.89)	0.641
Mixed	4.54 (1.29–15.91)	0.018	3.71 (0.91–15.29)	0.069
PD-L1				
Negative	1		1	
Positive	0.73 (0.38–1.42)	0.360	0.55 (0.27–1.10)	0.091
CD4/CD8				
Low	1		1	
High	0.35 (0.16–0.74)	0.006	0.27 (0.12–0.65)	0.004
LRP1B on TCs				
Negative	1		1	
Positive	0.43 (0.10–0.93)	0.042	0.54 (0.12–2.48)	0.432

GC, gastric cancer; DFS, disease-free survival; OS, overall survival; PD-L1, programmed cell death ligand 1; TCs, tumor cells.

## Data Availability

The original contributions presented in the study are included in the article. Further inquiries can be directed to the corresponding authors. The datasets presented in the study can be found in online repositories. The name of the repository/repositories and accession number can be found below: TCGA, STAD (https://portal.gdc.cancer.gov/projects/TCGA-STAD (accessed on 3 August 2021)). ClinicalTrials.gov NCT03607656. Registered on 1 July 2018. The final protocol version is V1.1.

## Supplementary Materials

The following supporting information can be downloaded at: https://www.mdpi.com/article/10.3390/cancers15245759/s1, Figure S1: Forest plot of univariate analysis in DEGs; Figure S2: The relationship between LRP1B expression on TCs/ICs and the ratio of TIIs in human gastric cancer tissue; Table S1: Pathological characteristics and high-frequency genes for patients in GC, Table S2: High- and low expression of 5 tumor infiltrating immune cells in 89 gastric cancer patients, Table S3: Univariate analysis for patients with GC.
