# Supplementary material for: Prognostic Implications of LRP1B and Its Relationship with the Tumor-Infiltrating Immune Cells in Gastric Cancer
Source: Cancers (Basel). 2023 Dec 8;15(24):5759. doi: 10.3390/cancers15245759 (PMC10741692; doi:10.3390/cancers15245759)
Supplement: Supplementary file 1 [file cancers-15-05759-s001.zip › Supplementary Figure Legends.pdf]

Supplementary Figure Legends:

Supplementary Figure S1: Forest plot of univariate analysis in DEGs. (DEGs: differentially expressed genes)

Supplementary Figure S2: The relationship between LRP1B expression on TCs/ICs and the ratio of TIIs in human gastric cancer tissue. (A) LRP1B expression on TCs with CD4/CD8. (B) LRP1B expression on TCs with CD86/CD163. (C) LRP1B expression on ICs with CD4/CD8. (D) LRP1B expression on ICs with CD86/CD163. (TCs: tumor cells; ICs: immune cells; TIIs: Tumor infiltrating immune cells)
