# Supplementary material for: Prognostic Implications of LRP1B and Its Relationship with the Tumor-Infiltrating Immune Cells in Gastric Cancer
Source: Cancers (Basel). 2023 Dec 8;15(24):5759. doi: 10.3390/cancers15245759 (PMC10741692; doi:10.3390/cancers15245759)
Supplement: Supplementary file 1 [file cancers-15-05759-s001.zip › Table S1.pdf]

Supplementary Table S1 Pathological characteristics and high-frequency genes for patients in GC

Parameters	N(%)	Parameters	N(%)	Parameters	N(%)	Parameters	N(%)
**HER2**		**PD-L1**		**KMT2D**		**RAD50**	
Negative	87(87.00)	Negative	47(47.00)	WT	82(82.00)	WT	95(95.00)
Positive	13(13.00)	Positive	53(53.00)	MUT	18(18.00)	MUT	5(5.00)
**Ki67**		**EBV**		**GLI3**		**HNF1A**	
＜50	24(24.00)	Negative	58(58.00)	WT	85(85.00)	WT	98(98.00)
≥50	67(67.00)	Positive	42(42.00)	MUT	15(15.00)	MUT	2(2.00)
NA [a]	9(9.00)	**TMB**		**ERBB3**		**TFE3**	
**P53**		TMB-L	68(68.00)	WT	72(72.00)	WT	72(72.00)
0	21(21.00)	TMB-H	32(32.00)	MUT	28(28.00)	MUT	28(28.00)
0.5	25(25.00)	**MSI**		**PIK3CA**		**FGFR2**	
1	39(39.00)	MSS	89(89.00)	WT	72(72.00)	WT	94(94.00)
NA	15(15.00)	MSI-H	11(11.00)	MUT	28(28.00)	MUT	6(6.00)
**Stat3**		**LRP1B**		**ACVR2A**			
Negative	9(9.00)	WT	77(77.00)	WT	89(89.00)		
Positive	83(83.00)	MUT	23(23.00)	MUT	11(11.00)		
NA	8(8.00)	**ARID1A**		**ERBB2**			
**PTBP3**		WT	72(72.00)	WT	89(89.00)		
Negative	7(7.00)	MUT	28(28.00)	MUT	11(11.00)		
Positive	85(85.00)	**TP53**		**TGFBR2**			
NA	8(8.00)	WT	37(37.00)	WT	90(90.00)		
**RUFY3**		MUT	63(63.00)	MUT	10(10.00)		
Negative	51(51.00)	**FAT4**		**PTEN**			
Positive	41(41.00)	WT	83(83.00)	WT	93(93.00)		
NA	8(8.00)	MUT	17(17.00)	MUT	7(7.00)		
**Bcl2**		**FAT3**		**TGFBR1**			
Negative	76(76.00)	WT	84(84.00)	WT	95(95.00)		
Positive	16(16.00)	MUT	16(16.00)	MUT	5(5.00)		
NA	8(8.00)						

N, number; NA, not available; HER-2, human epidermal growth factor receptor 2; stat3, signal

transducer and activator of transcription 3; PTBP3, Polypyrimidine tract binding protein 3; RUFY3, RUN and FYVE domain-containing protein 3; Bcl2, B cell lymphoma-2; PD-L1, programmed cell death ligand 1; EBA, Epstein-Barr virus; TMB, tumor mutation burden; MSS, microsatellite stability; MSI, microsatellite instability; WT, wild type; MUT, mutation type.
