# Supplementary material for: Prognostic Implications of LRP1B and Its Relationship with the Tumor-Infiltrating Immune Cells in Gastric Cancer
Source: Cancers (Basel). 2023 Dec 8;15(24):5759. doi: 10.3390/cancers15245759 (PMC10741692; doi:10.3390/cancers15245759)
Supplement: Supplementary file 1 [file cancers-15-05759-s001.zip › Table S2.pdf]

Supplementary Table S2 High- and low expression of 5 tumor infiltrating immune cells in 89 gastric cancer patients

**TIIs (median densities/HPF)**	**N**	**%**
**CD4**		
≤30.8/HPF	44	49.44%
＞30.8/HPF	45	50.56%
**CD8**		
≤49.6/HPF	42	47.19%
＞49.6/HPF	47	52.81%
**CD86**		
≤0.4/HPF	44	49.44%
＞0.4/HPF	45	50.56%
**CD163**		
≤19.4/HPF	44	49.44%
＞19.4/HPF	45	50.56%
**CD25**		
≤0/HPF	66	74.16%
＞0/HPF	23	25.84%

TIIs, tumor infiltrating immune cells; HPF, High-power field.
