# Supplementary material for: Prognostic Implications of LRP1B and Its Relationship with the Tumor-Infiltrating Immune Cells in Gastric Cancer
Source: Cancers (Basel). 2023 Dec 8;15(24):5759. doi: 10.3390/cancers15245759 (PMC10741692; doi:10.3390/cancers15245759)
Supplement: Supplementary file 1 [file cancers-15-05759-s001.zip › Table S3.pdf]

Supplementary Table S3 Univariate analysis for patients with GC

Parameters	DFS		OS	
	HR (95%CI)	P value	HR (95%CI)	P value
**Age**				
≥60 VS. < 60	0.89(0.45-1.75)	0.730	1.10(0.52-2.35)	0.806
**Tumor location**		0.186		0.441
Gastric body VS.EGJ/Cardia	0.23(0.03-1.80)	0.161	0.44(0.06-3.38)	0.428
Gastric antrum VS.EGJ/Cardia	0.37(0.05-1.83)	0.339	0.66(0.09-4.94)	0.688
**Histological type**		0.015		0.053
Undifferentiated VS. Differentiated	4.87(1.67-14.22)	0.004	4.40(1.30-14.83)	0.017
Mixed VS. Differentiated	1.11(0.50-2.43)	0.803	1.40(0.63-3.11)	0.415
**Lauren type**		0.367		0.417
Diffuse VS. Intestinal	1.45(0.75-2.82)	0.270	1.33(0.64-2.74)	0.443
Mixed VS. Intestinal	0.73(0.25-2.14)	0.569	0.58(0.17-1.95)	0.376
**p-TNM stage**				
III VS. II	2.30(0.82-6.48)	0.114	2.58(0.79-8.44)	0.117
**HER2**				
Positive VS. Negative	0.94(0.40-2.25)	0.897	0.89(0.35-2.30)	0.814
**PD-L1**				
Positive VS. Negative	0.51(0.27-0.95)	0.035	0.44(0.22-0.86)	0.016
**TMB**				
TMB-H VS. TMB-L	0.98(0.50-1.89)	0.941	0.84(0.40-1.75)	0.646
**Microsatellite status**				
MSI-H VS. MSS	0.63(0.19-2.03)	0.434	0.42(0.10-1.76)	0.235
**CD4/CD8**				
High VS. Low	0.28(0.14-0.57)	0.000	0.22(0.10-0.51)	0.000
**CD86/CD163**				
High VS. Low	1.11(0.60-2.07)	0.735	1.19(0.61-2.31)	0.609
**LRP1B on TCs**				
Positive VS. Negative	0.22(0.05-0.89)	0.034	0.25(0.06-0.99)	0.049
**LRP1B on ICs**				
Positive VS. Negative	0.91(0.65-1.28)	0.580	1.98(0.69-1.41)	0.925

GC, gastric cancer; DFS, disease free survival; OS, overall survival; HR, hazard ratio; CI, confident intervention; EGJ, gastroesophageal junction; p, pathological; HER-2; human epidermal growth factor receptor 2; PD-L1, programmed cell death ligand 1; TMB, tumor mutation burden; MSS, microsatellite stability; MSI, microsatellite instability.
