# Supplementary figures and images for: Prognostic Implications of LRP1B and Its Relationship with the Tumor-Infiltrating Immune Cells in Gastric Cancer
Source: Cancers (Basel). 2023 Dec 8;15(24):5759. doi: 10.3390/cancers15245759 (PMC10741692; doi:10.3390/cancers15245759)

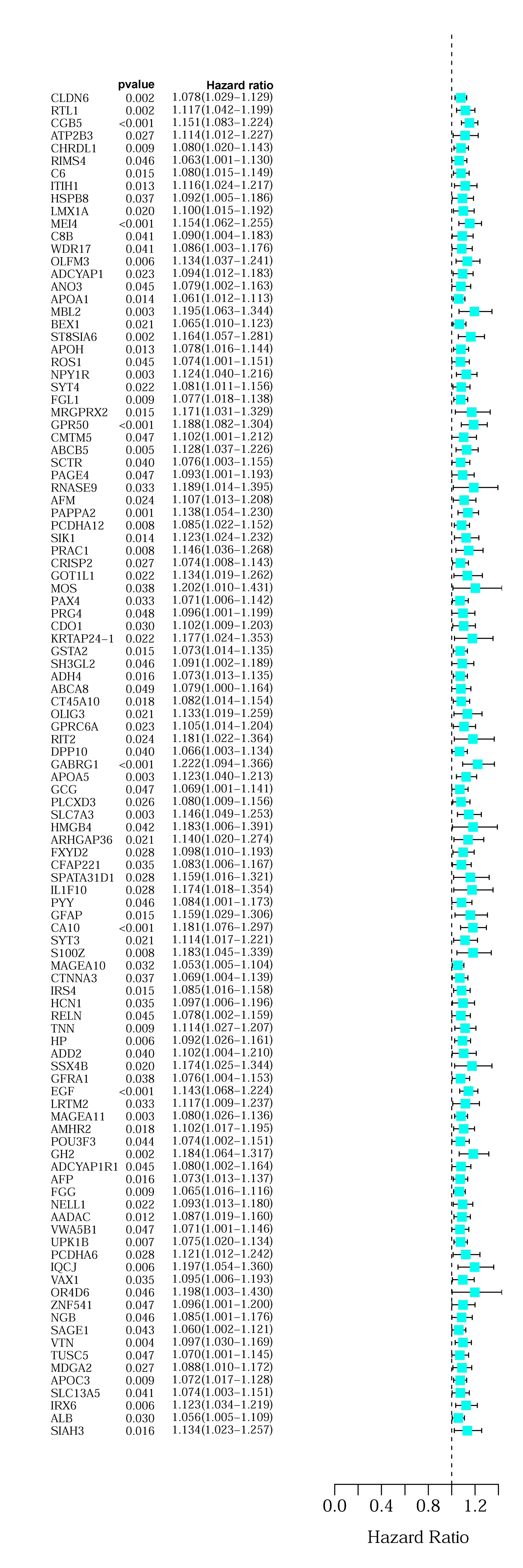

Supplement: Supplementary file 1 [file cancers-15-05759-s001.zip › Fig.S1.tif]

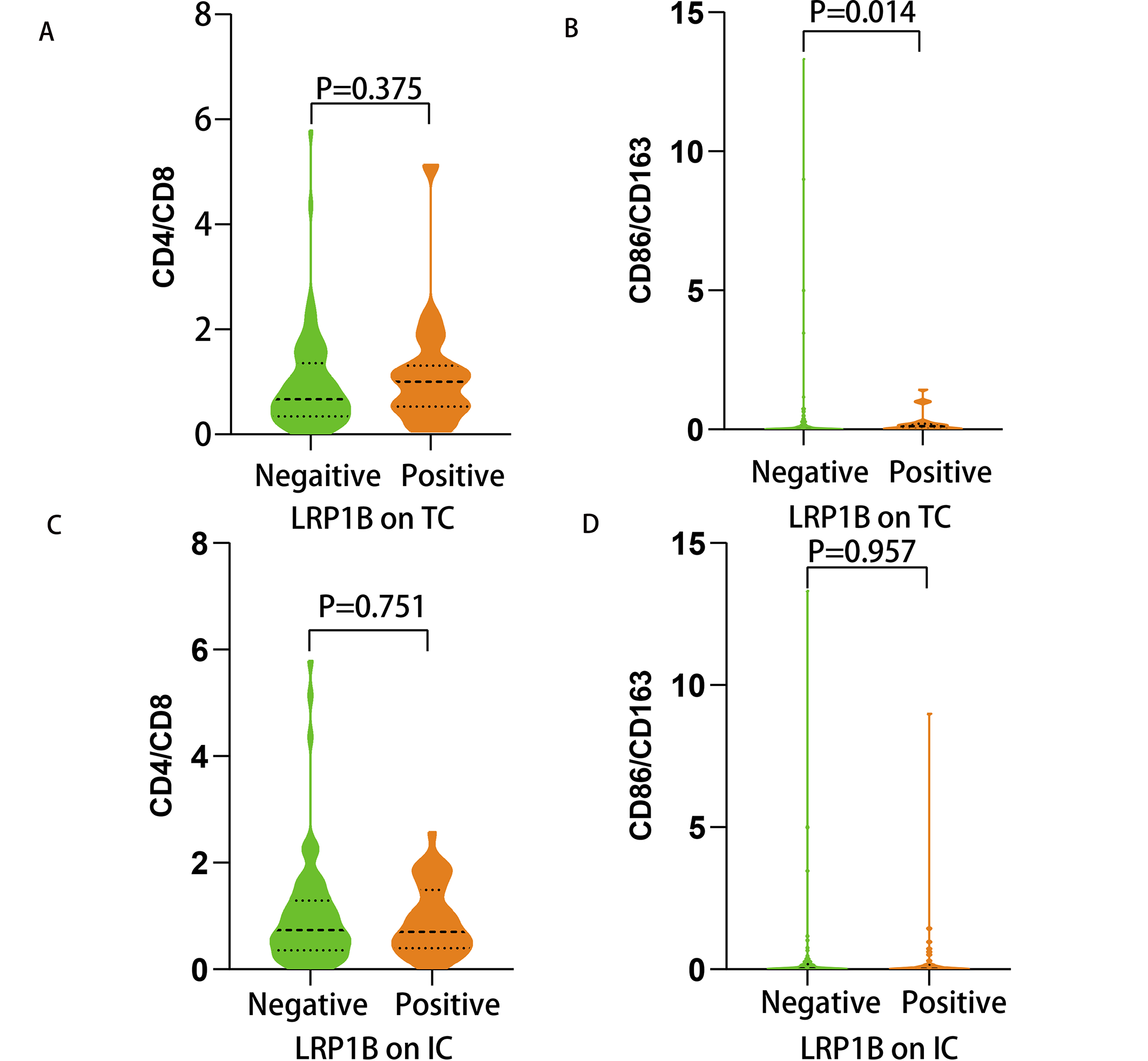

Supplement: Supplementary file 1 [file cancers-15-05759-s001.zip › Fig.S2.tif]
